# Molecular Characterization and Biological Function of a Novel LncRNA CRNG in Swine

**DOI:** 10.3389/fphar.2019.00539

**Published:** 2019-05-21

**Authors:** Qirong Lu, Li Li, Aixin Huang, Luqing Cui, Yinfeng Zhang, Qianying Liu, Xu Wang, Yulian Wang, Zhenli Liu, Zonghui Yuan, Menghong Dai

**Affiliations:** ^1^National Reference Laboratory of Veterinary Drug Residues and Ministry of Agriculture Key Laboratory for the Detection of Veterinary Drug Residues in Foods, Huazhong Agricultural University, Wuhan, China; ^2^Ministry of Agriculture Laboratory for Risk Assessment of Quality and Safety of Livestock and Poultry Products, Huazhong Agricultural University, Wuhan, China

**Keywords:** lncRNA CRNG, cyadox, immune, pathogen infection, inflammation

## Abstract

Our previous study has showed that a novel gene is differentially expressed in the liver of cyadox-fed piglets, but its sequence and function are unknown. Here, rapid amplification of cDNA ends (RACE) and bioinformatics analysis showed that the novel gene is 953 bp without protein-coding ability and locates in chromosome 11. Hence, we identified the novel gene as long non-coding RNA (lncRNA) and named it cyadox-related novel gene (CRNG). Fluorescence in situ hybridization (FISH) showed that CRNG mainly distributes in cytoplasm. Moreover, microarray assay in combination with CRNG interference and overexpression showed that the differential genes such as ANPEP, KITLG, STAT5A, FOXP3, miR-451, IL-2, IL-10, IL-6, and TNF-α are mainly involved in viral and pathogens infection and the immune-inflammatory responses in PK-15 cells. This work reveals that CRNG might play a role in preventing the host from being infected by pathogens and viruses and exerting immune regulatory effects in the cytoplasm, which may be involved in prophylaxis of cyadox in piglets.

## Introduction

Recent advances in high throughput sequencing technology have led to markedly expanding our knowledge of transcriptome, i.e., LncRNAs, which are longer than 200 nucleotides and unable to be translated into proteins (Li et al., 2014; Wu et al., 2014). The role of lncRNAs may far exceed people’s expectations, and increasing amount of evidence suggests that lncRNAs may play critical roles in control of a wide array of cellular functions, for example viral infection (Lemler et al., 2017), immune cell homeostasis and function (Chen et al., 2017; Mowel et al., 2018), inflammation (Chew et al., 2018), forcing us to radically attach importance to lncRNAs. In particular, attention is now shifting toward a type of RNA called LncRNAs, which is one of the most poorly understood, yet most common (Dinger et al., 2008; Kwok and Tay, 2017; Li et al., 2018).

Cyadox is a novel derivative of quinoxaline-1,4-dioxides with antibacterial and growth promotion effects (Cui et al., 2018; Guo et al., 2018) and with the potential to serve as a feed additive (Liu et al., 2018). A previous study in our lab has shown that eight differential genes were found through mRNA differential display technology in the liver of cyadox-fed piglets, including insulin-like growth factor-1, epidermal growth factor, poly ADP-ribose polymerase, the defender against apoptotic death 1, complement component 3, transketolase, *sus scrofa* zinc finger CCHC domain containing 3 and a novel gene (Yu et al., 2018). Additionally, the sequence of the novel gene is matched with a predicted sequence, *sus scrofa* uncharacterized LOC100626416, in nation center for biotechnology information, which was constantly updated in 2015, 2017, and 2018, and has not been experimentally demonstrated. Moreover, the novel gene was demonstrated without highly homologous protein sequences in protein data bank (Yu et al., 2018). Hence, we tentatively named the gene as CRNG. In the previous study, it was found that CRNG is related to the NF-kB, P38, TGF-β, JNK, PI3K, and JAK-STAT signaling pathway in primary cultured pig hepatocytes exposure to cyadox (Guo et al., 2018), suggesting that CRNG might be very important in cyadox action. Consequently, it is necessary to illustrate the full length, structural characteristics and biological function of CRNG, and further explain the role of the CRNG in the cyadox-mediated functional effects.

Considerable research efforts have been devoted to study the basic characterizations of the unknown gene via using the technology of RACE (Dieffenbach et al., 2003) to clone the full-length of novel gene, and bioinformatics, CPC (Kong et al., 2007) and ORF finder (Garcia et al., 2015), to analyze the ability of ending protein of novel genes. Additionally, RNA FISH was used to investigate the subcellular localization of lncRNAs to further elucidate the mechanisms and functions of lncRNAs (Huang et al., 2017; Das et al., 2018). Moreover, the combination of microarray and RT-qPCR can better analyze the properties and functions of genes (Shi et al., 2018).

This study aims at illustrating the characterization, function of CRNG in swine. The study on cyadox-related gene CRNG will help to provide a new sight on the pharmacological mechanism of cyadox. Our study showed that CRNG is a non-coding RNA mainly distributed in liver, followed by the jejunum and duodenum, and again the kidney of swine, and the cytoplasm of PK-15 cells. Microarray and RT-qPCR reveal important biological functions of CRNG, such as regulation of inflammation, pathogen infection and antiviral immunity, which provides a new view to better explain the development and application of cyadox and relevant immune mechanisms of CRNG in swine.

## Results

### Molecular Characteristics of LncRNA CRNG

**FIGURE 1:**
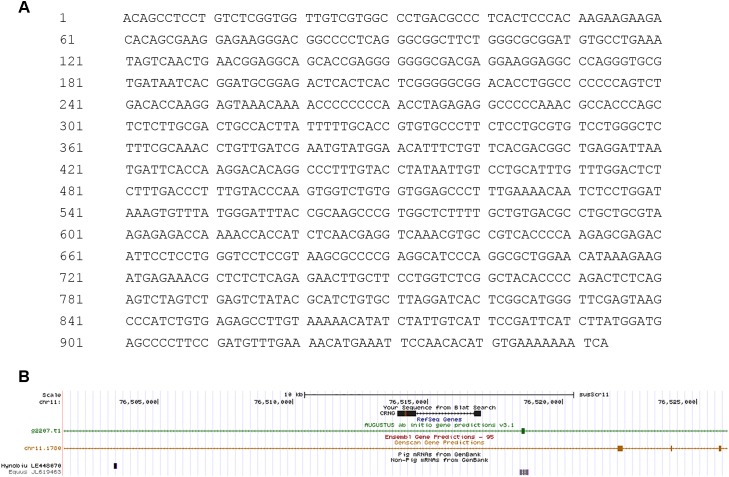
Schematic diagram of long non-coding RNA (lncRNA) cyadox-related novel gene (CRNG) sequence and structure. **(A)** The full length of CRNG is 953 bp; **(B)** CRNG consists of two exons (exon 1 and exon 2) and an intron in UCSC.

To explore the biological functions of CRNG, 953 bp of the full-length cDNA sequence of CRNG was obtained by 5′ and 3′ RACE (Figure 1A). Using BLAST searches for porcine HTGS database we obtained a partially match (GenBank NC-010453.5, 76514352–76516982) with the obtained porcine CRNG cDNA, which revealed that the genomic sequence of swine CRNG located in chromosome 11. Then CRNG sequence was searched in the whole genome of pigs, and the cDNA of which consists of exon 1 (76513882–76514572), exon 2 (76516721–76516981) and one intron (76514573–76516720). However, the exon 1 obtained by 5′ RACE is not full-match to the genomic sequence, which was verified by sequencing. It means that the CRNG sequence is one base “C” more than the genome sequence at 76514142 and base “C” replaces base “T” at 76514197 (Supplementary Data Sheet S1), which was further determined to be 953 bp by DNA amplification and sequencing (Figure 1B).

**FIGURE 2:**
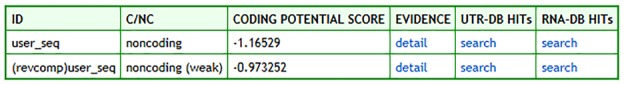
Coding ability prediction of CRNG full-length and its reverse complementary strand. The score of coding ability was minus, explaining that CRNG was a non-coding gene.

The ORF of CRNG was predicted by bioinformatics tools in NCBI^1^ (Table 2). The prediction results showed that the ORF has no coding capacity in porcine genome. Therefore, we also assessed the protein-coding potential of CRNG and its reverse complementary in a Support Vector Machine-based classifier: CPC^2^, it suggested that the gene CRNG was a non-coding RNA (Figure 2).

### Transcriptional Levels of CRNG in Different Tissues of Swine

**FIGURE 3:**
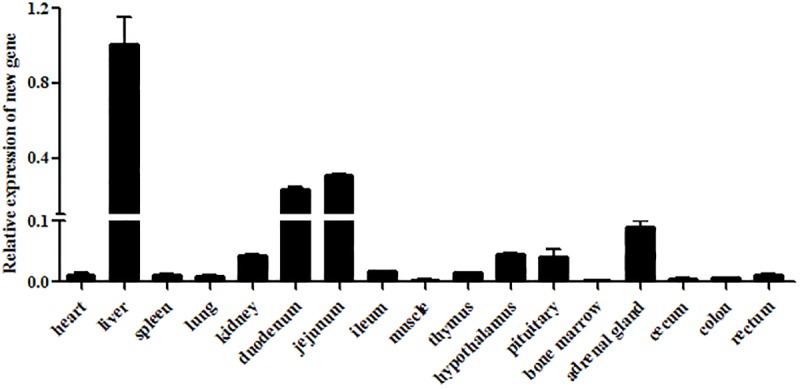
Relative expression levels of CRNG in different piglet tissues as measured by reverse transcription quantitative polymerase chain reaction (RT-qPCR). β-actin was used as a reference gene, liver was used as control. LncRNA CRNG was the highest in liver, high in duodenum and jejunum, low in kidney, adrenal gland, hypothalamus and pituitary, very low in other tissues. All experiments were performed in biological triplicates with three technical replicates.

The transcriptional levels of CRNG were evaluated relative to the endogenous β-actin mRNA levels in heart, liver, spleen, lung, kidney, duodenum, jejunum, ileum, longissimus dorsi muscle, thymus, hypothalamus, pituitary, bone marrow, adrenal gland, cecum, colon, rectum of swine by RT-qPCR. The result demonstrated that mRNA level of CRNG was the highest in liver, high in duodenum and jejunum, low in kidney, adrenal gland, hypothalamus and pituitary, very low in other tissues (Figure 3). In the pretest, the growth rate of the transfected IPEC-J2 cells slowed down, and there was a tendency to shed death, and the effect after transfection could not be detected. Therefore, PK-15 cells were finally selected as the cell line for the transfection experiment.

### Subcellular Localization of CRNG

**FIGURE 4:**
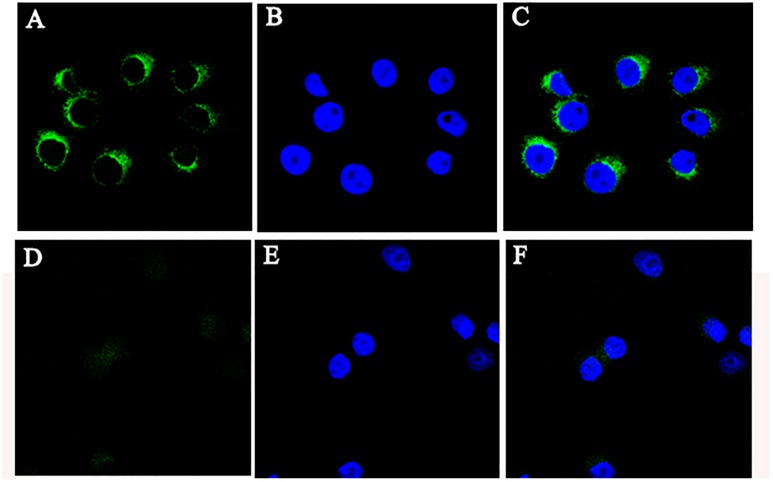
The location of CRNG RNA in PK-15 cells. **(A)** RNA fluorescence in situ hybridization (FISH) signal location using probes against CRNG; **(B)** The nucleus was stained with DAPI; **(C)** LncRNA CRNG predominantly is distributed in the cytoplasmic region in PK-15 cells; **(D–F)** RNA FISH showed probes were treated with RNase.

To gain insights into the precise mechanism of CRNG, we first examine its subcellular localization because the function of lncRNA depends on its subcellular distribution (Kopp and Mendell, 2018). FISH was used to observe the distribution of CRNG in PK-15 cells. As observed in Figure 4, CRNG predominantly is distributed in the cytoplasmic region of PK-15 cells. This finding provided the evidence that CRNG might act as endogenous sponge RNA to influence the expression of its target mRNA in cytoplasm.

### Global Differential Gene Expression in PK-15 Cells Interfering CRNG

**FIGURE 5:**
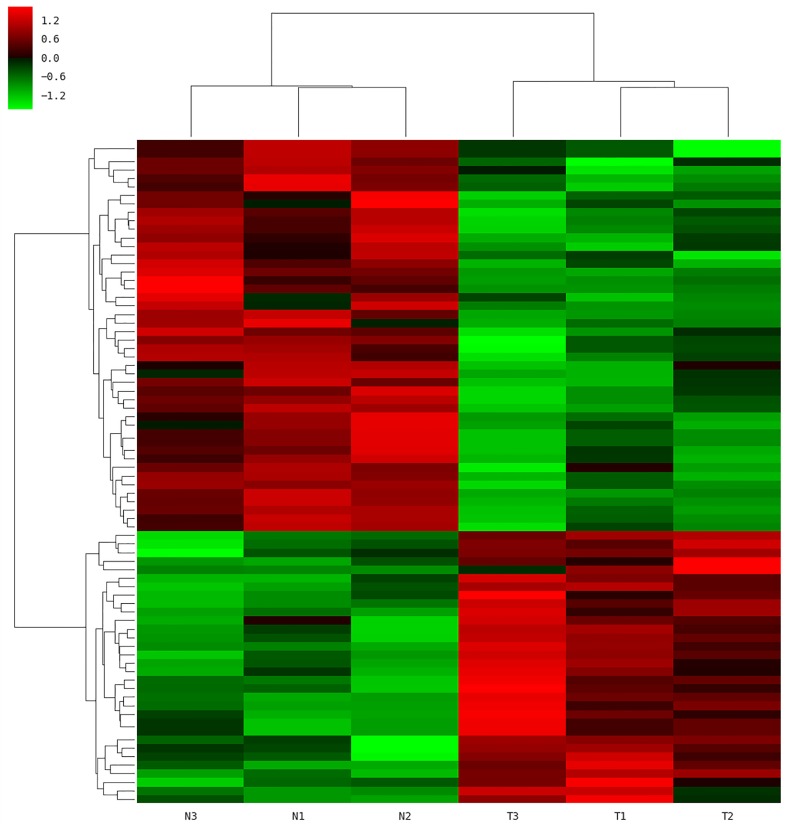
Microarray analysis was performed in PK-15 cells with siRNA-NC and siRNA CRNG. N1-3, control; T1-3, treated with siRNA against CRNG.

**FIGURE 6:**
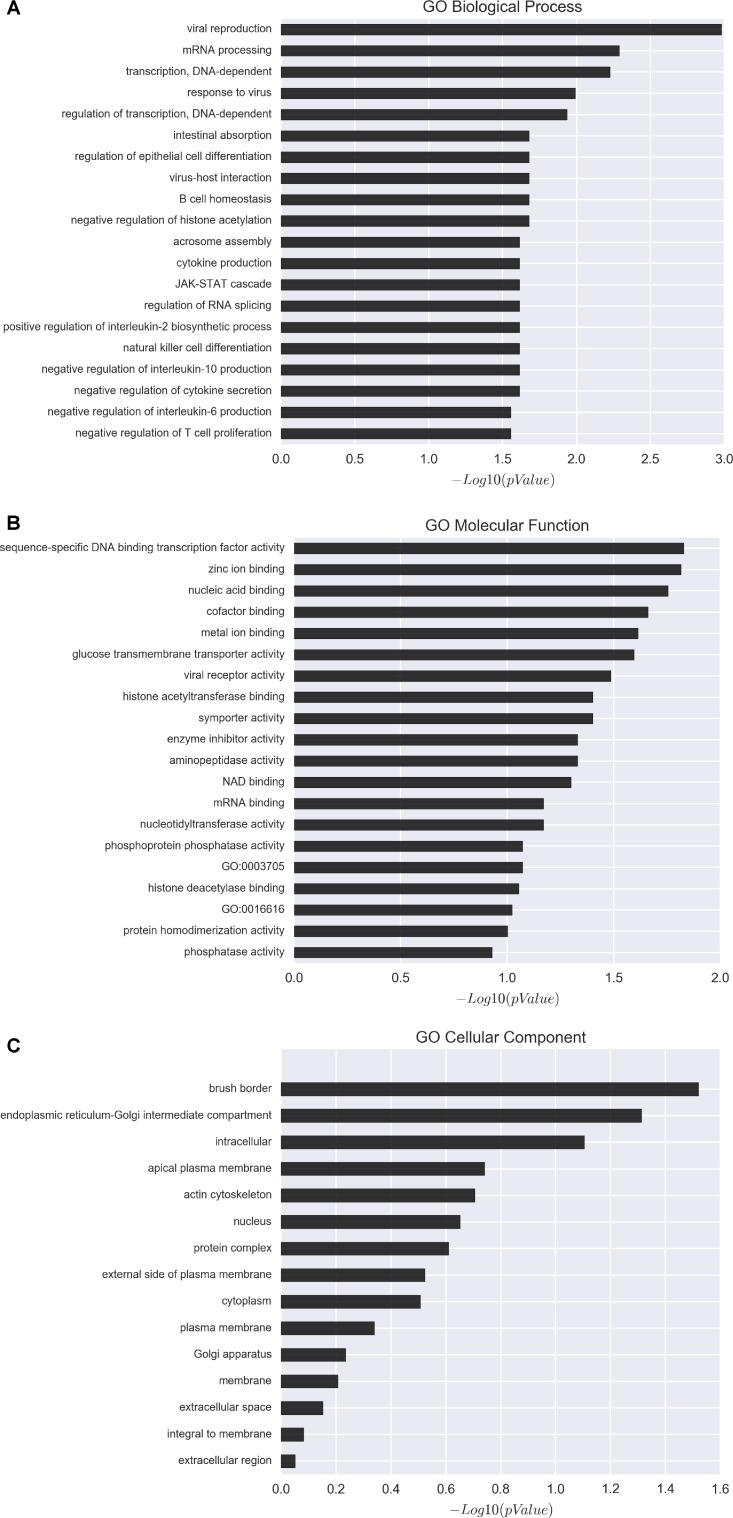
Gene ontology (GO) analysis of affected genes upon perturbation of CRNG functions via siRNAs. **(A)** GO biological process distribution of differentially expressed genes. **(B)** GO molecular function distribution of differentially expressed genes. **(C)** GO cellular component distribution of differentially expressed genes. GO-based enrichment analysis showed that the differentially expressed genes mainly enriched in immune and inflammation related pathway.

To gain further insight into how the manipulation of CRNG might modulate the function and biological process in PK-15 cells, we next determined the effects of siRNA-CRNG and siRNA-NC in PK-15 cells for 24 h by performing RNA-sequencing analysis. The statistical analysis showed that absence of the CRNG significantly affect the expression of 78 genes, including 32 upregulated genes and 46 downregulated genes. These gene sets were related to immune response pathways including interferon signaling, the cellular response to cytokine production, and viral process (Figure 5 and Supplementary Data Sheet S2). Additionally, the differential genes were individually annotated and elucidated the main biological process via GO-based enrichment analysis, including the cellular component, biological process and molecular function. Intriguingly, the analysis data of GO biological process confirmed that several biological pathways related to viral production, cytokine production, and inflammation were affected (Figure 6). These finding suggest that CRNG is an important part of immune and inflammation process.

### Validation of Differentially Expressed Genes in PK-15 Cells Overexpressing and Interfering CRNG

**FIGURE 7:**
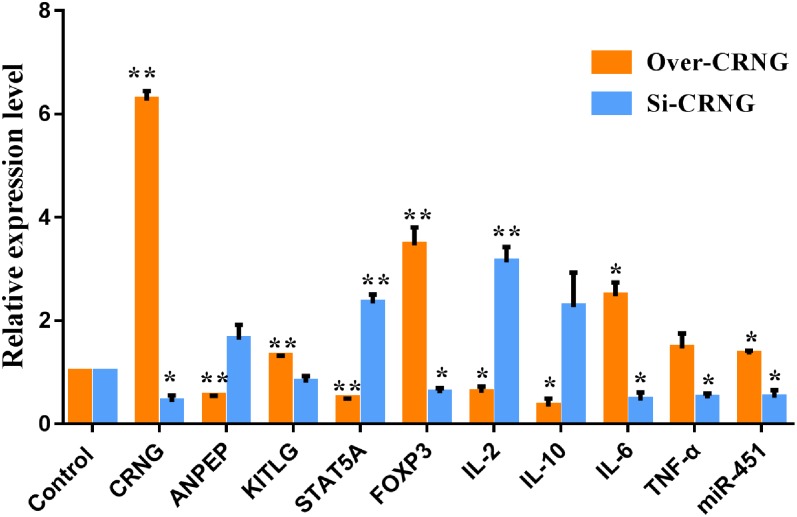
The mRNA and miRNA expression of differentially expressed genes after CRNG interference and overexpression in PK-15 cells. β-actin and U6 were used as reference gene for mRNA and miRNA. It means that CRNG could increase kit ligand (KITLG), forkhead box protein 3 (FOXP3), IL-6, TNF-α mRNA and miRNA-451 expression and inhibit alanyl aminopeptidase (ANPEP), signal transducer and activator of transcription 5A (STAT5A), IL-2 and IL-10 mRNA expression. Blocking CRNG could increase ANPEP, STAT5A, IL-2 and IL-10 mRNA and miRNA-451 expression and inhibit KITLG, FOXP3, IL-6, TNF-α mRNA expression. All experiments were performed in biological triplicates with three technical replicates. ^∗^*p* < 0.05, ^∗∗^*p* < 0.01.

To verify the reliability of the microarray assay results, several genes related to inflammation, infection and antiviral immunity, such as ANPEP, KITLG, STAT5A, FOXP3, and miR-451 were selected. Data indicated that CRNG significantly increase KITLG, FOXP3 and miR-451, and decrease ANPEP and STAT5A mRNA expression (Figure 7), suggesting that CRNG may be necessary for the modulation of antiviral immunity, inflammation and pathogen infection. Additionally, according to the GO analysis, several genes associated with inflammation, such as IL-2, IL-10, IL-6, TNF-α, were examined using RT-qPCR in PK-15 cells via overexpressing and interfering CRNG, respectively. The transcriptional levels of IL-2, IL-10, and IL-6 were decreased and increased in boosted-CRNG PK-15 cells, respectively, while that of TNF-α was unchanged (Figure 7). Intriguingly, in opposition to boosted-CRNG, siRNA-CRNG boosted IL-2 mRNA level and decreased IL-6 and TNF-α expression, while IL-10 was induced with no significance (Figure 7). These data indicate that CRNG directly modulate inflammatory response. Taken together, these results suggested that lncRNA CRNG might be a vital regulator of inflammation, pathogen infection and antiviral immunity.

## Materials and Methods

### Cell Line and Culture Conditions

The cryopreserved porcine kidney cell line PK-15 was purchased from American Type Culture Collection (ATCC, Rockville, MD, United States). The cryopreserved PK-15 was quickly thawed at 37°C in a water bath and centrifuged at low speed (50 *g*) for 5 min to remove DMSO, then suspended and cultured in Dulbecco’s modified Eagle’s medium/high glucose (HyClone, United States) containing 10% fetal bovine serum (Gibco, United States), 2 mM L-glutamine, 100 μg/mL streptomycin and 100 UI/mL penicillin. Cells were maintained in T-25 flasks at 37°C in a humidified 5% CO_2_ atmosphere with daily exchanges of fresh culture medium. When cells approached 100% confluence, cells were rinsed with PBS twice, and incubated with fresh 0.25% trypsin for 3 to 5 min, then sub-cultured at a ratio of 1:3 or 1:4 into new flasks. Cells were passaged three times prior to use in experiments.

### RT-qPCR

The RNA of PK-15 cells were lysed with Trizol^^®^^ Reagent (Invitrogen, United States) according to the instructions. The total RNA contaminating DNA was degraded by treating each sample with RNase-Free DNase Set (Sangon Biotech, China). After purification, total RNA was quantified by optical density (Q3000, Quawell, United States) and the quality was evaluated by gel electrophoresis. The first cDNA was synthesized by HiScript II 1st strand cDNA synthesis kit (Vazyme, China). All primers of target genes were commercially synthesized by Tianyi huiyuan biotechnology company, China (Table 1). The conditions of RT-qPCR were conducted by ChamQ SYBR Color qPCR Master Mix (Vazyme, China) according to the manufacturer’s instructions. The mRNA and miRNA levels were normalized against the amount of the housekeeping gene transcript β-actin and U6, respectively. And the relative expression was calculated by the 2^-ΔΔCt^ method (Wang X. et al., 2012).

**Table 1:** The list of primers sequences.

aa	Sequence (5′ to 3′)	Product
QT	CCAGTGAGCAGAGTGACGAGGACTCGAGCT	–
	CAAGCTTTTTTTTTTTTTTTTT
Q0	CCAGTGAGCAGAGTGACG	–
Q1	GAGGACTCGAGCTCAAGC	–
CRNG1	GTTTATGGGATTTACCGCAAGCC	–
CRNG2	CTCAACGAGGTCAAACGTGC	–
GSP1	AATGTCTCGCTCTTGGGGTG	–
GSP2	CAAAGGGCTCCACCACAGACCACTTG	–
GSP3	GAGTGAGTCTCCGCATCCGTGATTATC	–
CRNG	CTCAACGAGGTCAAACGTGC	219 bp
	TTACTCGAACCCATGCCGAG	
IL-10	CCTGACTGCCTCCCACTTTC	94 bp
	GGGCTCCCTAGTTTCTCTTCCT	
TNF-α	GCCCACGTTGTAGCCAATGTCAAA	99 bp
	GTTGTCTTTCAGCTTCACGCCGTT	
IL-6	AGATGCCAAAGGTGATGCCA	260 bp
	CCACAAGACCGGTGGTGATT	
IL-2	CATTGCACTAACCCTTGCACTC	81 bp
	GGCTCCAGTTGTTTCTTTGTGTT	
FOXP3	GGTGCAGTCTCTGGAACAAC	148 bp
	GGTGCCAGTGGCTACAATAC	
KITLG	GCAGGAACCGTGTGACTGAT	111 bp
	TAGGCAAAACGTCCATCCCG	
ANPEP	AAAGCATCGTCCGCTTACTCT	146 bp
	CAGCTCAGTCCTGTCGATCTC	
STAT5A	GTCCTGAAGACGCAGACCAA	316 bp
	ACTCGAACAGGACCGTGAAC	
miRNA-451 RT	CTCAACTGGTGTCGTGGAGTCGGCAATTCA	
	GTTGAGAACTCAGT
miRNA-451	ACACTCCAGCTGGGAAACCGTTACCATTAC	54 bp
	TGGTGTCGTGGAGTCG	
U6	CTCGCTTCGGCAGCACA	89 bp
	AACGCTTCACGAATTTGCGT	
β-actin	GCTGTCCCTGTACGCCTCTG	344 bp
	GCTCGTTGCCGATGGTGAT	


**Table 2:** ORF Finder of CRNG sequence.

ORF	Location	Length (nt | aa)	SmartBLAST
ORF7	557 – 880 (–)	324| 107	No SmartBLAST hits found
ORF6	417 – 713 (–)	297| 98	No SmartBLAST hits found
ORF1	550 – 792 (+)	243| 80	hypothetical protein [Chryseobacterium shigense]
ORF3	386 – 601 (+)	216| 71	hypothetical protein FIBSPDRAFT_938475 [Fibularhizoctonia sp. CBS 109695]
ORF8	203 – 394 (–)	192| 63	No SmartBLAST hits found
ORF4	420 – 524 (+)	105| 34	Granzyme K, partial [Anas platyrhynchos]
ORF2	826 – 927 (+)	102| 33	ompA family protein, partial [Acinetobacter baumannii 45057_1]
ORF5	795 – 890 (–)	96| 31	hypothetical protein [Haloferax prahovense]


### Expression Profile of CRNG Gene

Seventeen porcine tissues, i.e., heart, liver, spleen, lung, kidney, duodenum, jejunum, ileum, longissimus dorsi muscle, thymus, hypothalamus, pituitary, bone marrow, adrenal gland, cecum, colon, and rectum, were collected from three Landrace × Large White crossbred barrows in age from 5 to 6 weeks from the pig farm of Huazhong Agricultural University. The current study was approved by the Ethical Committee of the Faculty of Veterinary Medicine (Huazhong Agricultural University). All the tissue RNAs were extracted completely according to the EZ-10 total RNA mini-preps kit instructions (Sangon Biotech, China). β-actin gene was used as an internal control. The expression profile and internal control primers were designed according to the CRNG sequence (Table 1). Analysis of relative gene expression was conducted using RT-qPCR and the 2^-ΔΔCt^ method (Wang X. et al., 2012).

### 3′ RACE and 5′ RACE PCR

The 3′ RACE and 5′ RACE experiment was performed in full accordance with the classical RACE method of PCR Primer: a laboratory manual (Dieffenbach et al., 2003). For 3′ RACE, the first cDNA was synthesized by reverse transcriptase M-MLV (RNase H-; Takara, Japan) with Q_T_, then the ribonuclease H (Beyotime, China) was utilized to remove the RNA from cDNA. Simultaneously the cDNA was purified by SanPrep column PCR product purification kit (Sangon Biotech, China). The outer PCR was carried out with gene specific primer CRNG1 and Q_0_ via the phanta max super-fidelity DNA polymerase (Vazyme, China). And the inner PCR was carried out with gene specific primer CRNG2 and Q_1_ via taq DNA Polymerase (Mg^2+^ Plus Buffer; Vazyme, China). For 5′ RACE, the first cDNA was synthesized by HiScript II 1st strand cDNA synthesis kit (Vazyme, China) with gene specific primer GSP1, then the ribonuclease H (Beyotime, China) was utilized to remove the RNA from cDNA. And the poly “A” tail was added at the end of cDNA by dATP and terminal deoxynucleotidyl transferase (Takara, Japan). Then the cDNA was purified by SanPrep column PCR product purification kit (Sangon Biotech, China). The outer PCR was carried out with gene specific primer GSP2, Q_0_ and Q_T_ via the phanta max super-fidelity DNA polymerase (Vazyme, China), and the inner PCR was carried out with gene specific primer GSP3 and Q_1_ via taq DNA Polymerase (Mg^2+^ Plus Buffer; Vazyme, China). All the PCR products were analyzed by 1% agarose gel and sequenced by TA cloning (Tianyi huiyuan, China). All the primers were summarized in Table 1.

### LncRNA – CRNG Identification

The full-length cDNA of CRNG was obtained via RACE. Simultaneously, for identifying the characteristic of CRNG, genome location and coding potential were analyzed via NCBI Genome and ORF Finder (Zhang et al., 2012), CPC (Kong et al., 2007), respectively. Additionally, the ORF finder can search novel DNA sequence for potential protein encoding fragments, then verify predicted protein via SMART BLAST or regular BLASTP. No homologous protein sequence of the result of ORF Finder and the score of CPC < 0 were used to assess the coding potential of transcripts. Then combine the results of the above coding ability analysis software to determine the coding ability of lncRNA.

### RNA FISH

RNA FISH was performed based on the protocol in the *Regulatory Non-Coding RNAs* (Carmichael, 2015) with some adjustments. A FITC-Oligo nucleotide probe was designed and synthesized by Shanghai Bogoo Bio-Technique Co., Ltd., for the target gene CRNG: 5′-GGTGACGGCACGTTTGACCTCGTTGAGATGGTG-3′. The specificity of the probe sequence was detected in the NCBI database. Cultured cells were seeded in the 6-wells of chamber slides and exactly fixed by paraformaldehyde. The slides were pretreated for hybridization by a 0.01 % proteinase K digestion (20 min, 37°C) in 0.01 M HCl, followed by a short wash in 0.1 M Glycine Irrigation. Then, the slides were fixed in 4% formaldehyde for 10 min at room temperature. The slides were washed in PBS three times. The slides were washed with acetic anhydride pH = 8.0 (acetylation, reduce the background) at room temperature, 5 min, 2 times. The slides were washed in PBS two times, and then washed in 5 × SSC (pH = 7.5), 1 min, 2 times. The slides were covered with the hybridization solution and incubated in a humidified chamber for 1 h at 65°C. The hybridization mixture contained 500 ng/mL of FITC-probe cover the slides in hybridization instrument at 62°C, 72 h. The slides were washed in 2 × SSC (pH = 7.5), at room temperature, 1 min, 1 time and washed in PBS two times. The slides were stained with DAPI, mounted with anti-quenching agent, and then observed under a fluorescent microscope (IX-71, Olympus, Japan). RNA specificity was confirmed by destruction of signals when samples were pretreated with RNase.

### Overexpression and Interference of LncRNA-CRNG

The over-expression plasmid pcDNA3.0-EGFP-CRNG was constructed by Tianyi huiyuan biotechnology company, China. siRNA2 for lncRNA-CRNG (sense 5′-GGACUCUCUUUGACCCUUUTT-3′; antisense 5′-AAAGGGUCAAAGAGAGUCCTT-3′) and a negative control siNC (sense 5′-UUCUCCGAACGGUCACGUTT-3′; antisense 5′-ACGUGACACGUUCGGAGAATT-3′) were obtained from GenePharma, Shanghai, China. The PK-15 cell transfection of the over-expression plasmid was conducted by ExFect^^®^^2000 Transfection Reagent (Vazyme, China) according to the manufacturer’s instructions. The PK-15 cell transfection of siRNA was conducted by Lipofectamine^TM^ 2000 Transfection Reagent (Invitrogen, United States) according to the manufacturer’s instructions. The efficiency of overexpression or interference was determined by RT-qPCR. Moreover, the siRNA sequence of CRNG and the silencing or overexpression efficiency after transfecting the cells with siRNA or CRNG-expression vector were shown in Supplementary Data Sheet S3.

### Microarray Analysis

The genechip was performed by Oebiotech Company (Shanghai, China). Total RNA of siRNA was quantified by the NanoDrop ND-2000 (Thermo Scientific) and the RNA integrity was assessed using Agilent Bioanalyzer 2100 (Agilent Technologies). The sample labeling, microarray hybridization and washing were performed based on the manufacturer’s standard protocols. Briefly, total RNAs were transcribed to double strand cDNAs and then synthesized cRNAs. Next, 2nd cycle cDNAs were synthesized from cRNAs. Followed fragmentation and biotin labeling, the 2nd cycle cDNAs were hybridized onto the microarray. After washing and staining, the arrays were scanned by the Affymetrix Scanner 3000 (Affymetrix). Affymetrix GeneChip Command Console (version 4.0, Affymetrix) software was used to extract raw data. Next, Expression Console (version1.3.1, Affymetrix) software offered RMA normalization for gene. Then the gene expression analysis was carried out.

### Statistical Analysis

All data were presented as mean ± SD, and statistical analyses were analyzed by PASW Statistics 18 software. Only *p* < 0.05 was considered significant. “^∗^” indicates *p*< 0.05; “^∗∗^” indicates *p*< 0.01. GeneSpring software (version 13.1; Agilent Technologies) was employed to finish the basic analysis. Differentially expressed genes were then identified through fold change as well as *P*-value calculated with *t*-test. The threshold set for up- and down-regulated genes was a fold change > = 1.5 and a *P*-value < = 0.05. Afterward, GO analysis was applied to determine the roles of these differentially expressed mRNAs played in these GO terms. Finally, Hierarchical Clustering was performed to display the distinguishable genes’ expression pattern among samples.

## Discussion

Long non-coding RNAs, non-coding transcripts of more than 200 nucleotides, are becoming key regulators for the expression and biological processes of some genes (Esteller, 2011). Based on previous study, we firstly clone the full-length sequence of CRNG, which consists of 953 bp without protein-coding ability. Moreover, the distribution of tissue expression profiles suggested that CRNG may play an important role in regulating liver and small intestine development and maintaining their functions. Our results indicate that CRNG is a long-non-coding RNA and might involve in various biological process in the development of pigs. Additionally, RNA FISH revealed that CRNG was predominantly located in the cytoplasm, indicating CRNG might serve as a competing endogenous RNA to sponge miRNAs and restore mRNA translation (Wang et al., 2014), or form complexes with diverse structural and regulatory functions to control mRNA turnover, translation, protein stability, sponging of cytosolic factors, and modulation of signaling pathways (Noh et al., 2018).

Accumulating studies have demonstrated the importance of lncRNAs in the regulation of immune and inflammatory responses (Atianand and Fitzgerald, 2014; Fitzgerald and Caffrey, 2014; Elling et al., 2016). One of the functional studies revealed that lincRNA-Cox2 broadly regulated the expression of a large number of immune genes and inflammatory response genes including pro-inflammatory cytokines and other inflammatory mediators (Carpenter et al., 2013). Previous study illustrated that after exposure to cyadox at the final concentration of 2 μM for 0.5, 1, 2, 4, and 8 h, CRNG mRNA expression would significantly increase in porcine primary hepatocytes compared with that of the control at 1 h. Moreover, the signaling pathways also showed that inflammation-related signaling pathways such as JNK and NF-κB, were involved in the regulation of CRNG (Guo et al., 2018). CRNG mRNA could also be activated by NF-κB, JAK-STAT and JNK pathways in cyadox-treated PK-15 cells (data not published). Present study showed that lncRNA CRNG predominantly associated with viral reproduction, response to virus, pathogen infection and the immune-inflammatory responses through regulating the genes of inflammation, pathogen infection and antiviral immunity, such as ANPEP, KITLG, STAT5A, FOXP3, miR-451 and immune and inflammatory factors, such as IL-2, IL-10, IL-6, TNF-α. Our study revealed that the CRNG has strongly relationship with immune, inflammation and pathogen infection.

ANPEP (also known as CD13) is a membrane metalloprotease consisting of 150 kDa, and an ectoenzyme of zinc-dependent aminopeptidases (Reinhold et al., 2007; Nefla et al., 2015). Recent studies have illustrated that ANPEP could involve in many biological process, such as the regulation of antigen-presenting (Thomas et al., 1994), immunoregulation (Biton et al., 2006), intestinal cholesterol absorption (Kramer et al., 2005). The compound of human CD13 and antibody could inhibit infection and block binding of HCMV virions to susceptible cells (Soderberg et al., 1993). In present study, we found that lncRNA CRNG could negatively regulate the expression of ANPEP, in addition, GO biological process had shown that lncRNA CRNG may involve in virus-host interaction. APN/CD13 could also serve as a modulator to T cell and target of tissue-specific autoimmunity in the CNS (Biton et al., 2006). CD13 served as a negative regulator for activation of mast cells *in vitro* and *in vivo* (Metz and Maurer, 2007; Ghosh et al., 2012). The lack of CD13 would activate the inflammation pathways, such as IL-6 and TNF-α, in a Fc𝜀RI-dependent manner (Zotz et al., 2016). From our data, it is clear that the absence of CRNG increased CD13 expression and inhibited the expression of pro-inflammatory factor, IL-6 and TNF-α, suggesting that CRNG may depend on CD13 signaling pathway involved in the regulation of inflammatory cytokines. These results indicated that lncRNA CRNG could inhibit the expression of CD13, thus activating inflammatory immune pathway and preventing the virus infection.

Kit ligand also called mast cell growth factor SCF or c-kit ligand, which is the main survival and developmental factor for mast cells (Ceponis et al., 1998). The combination of hyper-IL-6, SCF and GM-CSF could promote the differentiation of dendritic cells, thus stimulating the resting T cells against the processed antigen (Bernhard et al., 2000). Furthermore, IL-6 and SCF could partly promote the maturation of human cultured mast cells, and other factors may be involved in this process (Matsushima et al., 2000). In this paper, IL-6 and SCF had the same up-regulated trends, when lncRNA CRNG was overexpressed. It demonstrated that lncRNA CRNG may oppose antigen through the network of IL-6 and SCF.

The cellular responses to cytokines, such as SCF and interferons, depend on prior activation of the JAK/STAT signaling pathway. STAT5A showed a strong connection with immunity and virus production in primary CD T cells (Selliah et al., 2006). Notably, SCF not only participates in the regulation of inflammatory factors but also regulates the immunity-regulation through the activity of STAT5, which was reduced in G-CSF or SCF stimulated PNH clone cells (Ding et al., 2012).Additionally, IL-2 also could activate STAT5 and then inhibit the binding of STAT3 to IL17 locus, thus determining the extent of T_H_17 cell generation (Yang et al., 2011). Splenocytes from STAT5A-knockout mice showed the partial impairment in IL-2-induced proliferation and defected proliferation in T-lymphocyte and NK-cell (Nakajima et al., 1997; Moriggl et al., 1999). Present study revealed that boosted lncRNA CRNG could decrease the levels of IL-2 and STAT5A, and increase the level of KITLG and IL-6. A reasonable inference can be drawn that lncRNA CRNG could inhibit the activity of STAT5A, and thereby promote inflammation release and reduce virus production, thus revealing the pharmacological activity of cyadox.

FOXP3 is a transcriptional regulators, a member of the forkhead/winged-helix family (Triulzi et al., 2013), implicated in the regulation of the development and inhibitory function of T-cells (Treg), serving as a mediators for self-tolerance, immune homeostasis and various inflammatory responses (Bin Dhuban et al., 2014). The mutation of FOXP3 would lead to the loss of immune homeostasis in mice and humans (Gambineri et al., 2003; Kasprowicz et al., 2003). Moreover, ectopic expression of FOXP3 repressed the production of IL-2 in conventional T cells, through interacting physically with AML1 (Ono et al., 2007), thus controlling the physiological and pathological immune responses mediated by T cell. Additionally, Foxp3+ cells might have a role in the pathogenesis of active periodontal lesions through repressing the expression of TGF-β1 and IL-10 (Scott et al., 2009). They are in agreement with our results performed in PK-15 cells demonstrating that lncRNA CRNG could increase IL-6,TNF-α, FOXP3 mRNA expression, and inhibit IL-10 mRNA expression, thus suppressing viral reproduction and preventing immune hyperactivity.

Evidence that host and pathogens interact through miRNA pathways was documented in mammalian infectious diseases (Roberts et al., 2011; Liang et al., 2013). Wang et al. illustrated that miR-451 could markedly reduce the *var* gene of parasite virulence factor *P. falciparum* erythrocyte membrane protein-1 (Wang et al., 2017). miR-451 also was associated with autoimmune disease, which was increased in serum levels (Wang H. et al., 2012; Yamada et al., 2014). Notably, increased levels of IL-6 and IFN-β can positively regulate miR-451 expression, and miR-451 expression ultimately leads to a decrease in IL-6 expression, which in turn buffers IL-6 expression (Rosenberger et al., 2012). Our study also showed that lncRNA CRNG could enhance miR-451 expression, thus protecting host from pathogens infection. Meanwhile, lncRNA CRNG can also increase the expression level of IL-6, but whether CRNG is involved in the regulation of IL-6 through miR-451 remains to be further studied.

**FIGURE 8:**
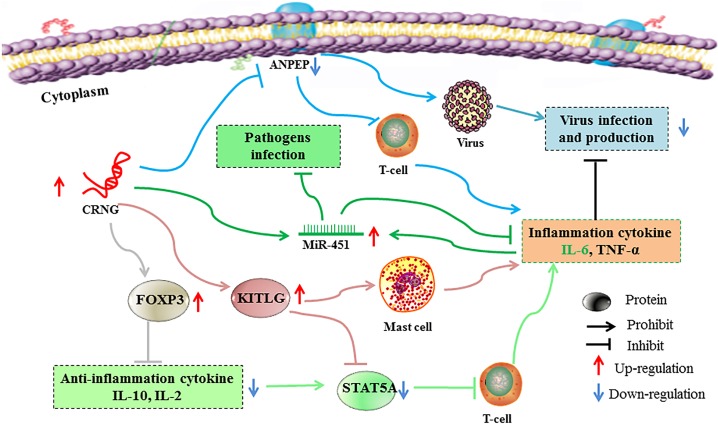
Possible mechanisms of lncRNA regulating immunity and inflammation. Boosted lncRNA CRNG could increase FOXP3 and KITLG expression and inhibit ANPEP and STAT5A expression, thus promoting pro-inflammation cytokine and suppressing anti-inflammation cytokine and virus infection and production. Moreover, CRNG could enhance the expression of miR-451, thus protecting host from pathogens infection, and indirectly regulate the homeostatic of IL-6 levels. Each color line represents a signal pathway. ANPEP, Alanyl aminopeptidase, membrane; CRNG, Cyadox-related novel gene; FOXP3, Forkhead box protein 3; KITLG, Kit ligand; miR-451, microRNA 451, STAT5A: Signal transducer and activator of transcription 5A.

Collectively, the molecular characterization and biological function of the novel gene lncRNA CRNG was initially analyzed for the first time. A reasonable conjecture is concluded that lncRNA CRNG could participate in several viral reproductions, responding to virus, infection and immune-inflammation through regulating the genes expression of inflammation and antiviral immunity, including ANPEP, KITLG, STAT5A, FOXP3, miR-451 and immune and inflammatory factors, IL-2, IL-10, IL-6, TNF-α (Figure 8). However, the mechanisms of lncRNA CRNG coupled with those related genes should be further investigated, in order to provide the alternative strategy for preventing the virus replication and further reveal the molecular mechanism of cyadox.

## Ethics Statement

The current study was approved by the Ethical Committee of the Faculty of Veterinary Medicine (Huazhong Agricultural University).

## Author Contributions

QLu, LL, and AH generated, analyzed, and interpreted the data, and prepared the manuscript. LC, YZ, QLi, XW, YW, and ZL generated the format and grammar modification. ZY and MD generated the idea, designed the study, analyzed and interpreted the data, and wrote the manuscript.

## Conflict of Interest Statement

The authors declare that the research was conducted in the absence of any commercial or financial relationships that could be construed as a potential conflict of interest.

## Footnotes

1 https://www.ncbi.nlm.nih.gov/orffinder/

2 http://cpc.cbi.pku.edu.cn

## Supplementary Material

DATA SHEET S1 The result of comparing CRNG with NCBI database.

DATA SHEET S2 The expressions of 78 genes upon the interfered CDRG via siRNA2, including 32 upregulated genes and 46 downregulated genes, show in excel.

DATA SHEET S3 The silencing or overexpression efficiency after transfecting the cells with siRNA or CRNG- expression vector, the silencing efficiency of CRNG was showed in Figure 2 and CRNG induced by cyadox in PK-15 cells was shown in Figure 3. The siRNA sequences of CRNG were showed in Table 1.

## Abbreviations

ANPEP: alanyl aminopeptidase
CPC: coding potential calculator
CRNG: cyadox-related novel gene
FISH: fluorescence in situ hybridization
FOXP3: forkhead box protein 3
GO: gene ontology
KITLG: kit ligand
LncRNA: long non-coding RNA
MiR-451: MicroRNA 451
ORF: open reading frame
RACE: rapid amplification of cDNA ends
RT-qPCR: reverse transcription quantitative polymerase chain reaction
SCF: stem cell factor
STAT5A: signal transducer and activator of transcription 5A
Treg: regulatory T-cells

## References

[B1] Atianand M. K. Fitzgerald K. A. (2014). Long non-coding RNAs and control of gene expression in the immune system. *Trends Mol. Med.* 20 623–631. 10.1016/j.molmed.2014.09.002 25262537 PMC4252818

[B2] Bernhard H. Lohmann M. Batten W. Y. Metzger J. Lohr H. F. Peschel C. (2000). The gp130-stimulating designer cytokine hyper-IL-6 promotes the expansion of human hematopoietic progenitor cells capable to differentiate into functional dendritic cells. *Exp. Hematol.* 28 365–372. 10781894 10.1016/s0301-472x(00)00126-0

[B3] Bin Dhuban K. Kornete M. Mason E. S. Piccirillo C. A. (2014). Functional dynamics of Foxp3(+) regulatory T cells in mice and humans. *Immunol. Rev.* 259 140–158. 10.1111/imr.12168 24712464

[B4] Biton A. Bank U. Tager M. Ansorge S. Reinhold D. Lendeckel U. (2006). Dipeptidyl peptidase IV (DP IV, CD26) and aminopeptidase N (APN, CD13) as regulators of T cell function and targets of immunotherapy in CNS inflammation. *Adv. Exp. Med. Biol.* 575 177–186. 10.1007/0-387-32824-6_19 16700521

[B5] Carmichael G. G. (2015). *Regulatory Non-Coding RNAs. Methods in Molecular Biology.* New York, NY: Humana Press.

[B6] Carpenter S. Aiello D. Atianand M. K. Ricci E. P. Gandhi P. Hall L. L. (2013). A long noncoding RNA mediates both activation and repression of immune response genes. *Science* 341 789–792. 10.1126/science.1240925 23907535 PMC4376668

[B7] Ceponis A. Konttinen Y. T. Takagi M. Xu J. W. Sorsa T. Matucci-Cerinic M. (1998). Expression of stem cell factor (SCF) and SCF receptor (c-kit) in synovial membrane in arthritis: correlation with synovial mast cell hyperplasia and inflammation. *J. Rheumatol.* 25 2304–2314. 9858422

[B8] Chen Y. G. Satpathy A. T. Chang H. Y. (2017). Gene regulation in the immune system by long noncoding RNAs. *Nat. Immunol.* 18 962–972. 10.1038/ni.3771 28829444 PMC9830650

[B9] Chew C. L. Conos S. A. Unal B. Tergaonkar V. (2018). Noncoding RNAs: master Regulators Of Inflammatory Signaling. *Trends Mol. Med.* 24 66–84. 10.1016/j.molmed.2017.11.003 29246760

[B10] Cui L. Xing D. Huang D. Li D. Lu Q. Wang X. (2018). Signaling pathways involved in the expression of SZNF and the target genes binding with SZNF related to cyadox. *Biomed. Pharmacother.* 108 1879–1893. 10.1016/j.biopha.2018.09.141 30453449

[B11] Das S. Zhang E. Senapati P. Amaram V. Reddy M. A. Stapleton K. (2018). A novel angiotensin II-induced long noncoding rna giver regulates oxidative stress, inflammation, and proliferation in vascular smooth muscle cells. *Circ. Res.* 123 1298–1312. 10.1161/circresaha.118.313207 30566058 PMC6309807

[B12] Dieffenbach C. W. Dveksler G. S. Dieffenbach C. W. Dveksler G. S. (2003). *PCR Primer: A Laboratory Manual.* New York, NY: Cold Spring Harbor Laboratory Press.

[B13] Ding S. X. Fu R. Wang H. L. Zhang T. Ruan E. B. Qu W. (2012). Expression of phosphorylated STAT5 in bone marrow hematopoietic stem cells of patients with paroxysmal nocturnal hemoglobinuria before and after in vitro G-CSF or SCF stimulation. *Zhonghua Yi Xue Za Zhi* 92 956–959. 22781567

[B14] Dinger M. E. Amaral P. P. Mercer T. R. Pang K. C. Bruce S. J. Gardiner B. B. (2008). Long noncoding RNAs in mouse embryonic stem cell pluripotency and differentiation. *Genome Res.* 18 1433–1445. 10.1101/gr.078378.108 18562676 PMC2527704

[B15] Elling R. Chan J. Fitzgerald K. A. (2016). Emerging role of long noncoding RNAs as regulators of innate immune cell development and inflammatory gene expression. *Eur. J. Immunol.* 46 504–512. 10.1002/eji.201444558 26820238 PMC5404502

[B16] Esteller M. (2011). Non-coding RNAs in human disease. *Nat. Rev. Genet.* 12 861–874. 10.1038/nrg3074 22094949

[B17] Fitzgerald K. A. Caffrey D. R. (2014). Long noncoding RNAs in innate and adaptive immunity. *Curr. Opin. Immunol.* 26 140–146. 10.1016/j.coi.2013.12.001 24556411 PMC3932021

[B18] Gambineri E. Torgerson T. R. Ochs H. D. (2003). Immune dysregulation, polyendocrinopathy, enteropathy, and X-linked inheritance (IPEX), a syndrome of systemic autoimmunity caused by mutations of FOXP3 a critical regulator of T-cell homeostasis. *Curr. Opin. Rheumatol.* 15 430–435. 12819471 10.1097/00002281-200307000-00010

[B19] Garcia R. A. Pereira M. R. Maester T. C. De Macedo Lemos E. G. (2015). Investigation, expression, and molecular modeling of ORF2 a metagenomic lipolytic enzyme. *Appl. Biochem. Biotechnol.* 175 3875–3887. 10.1007/s12010-015-1556-8 25764223

[B20] Ghosh M. Mcauliffe B. Subramani J. Basu S. Shapiro L. H. (2012). CD13 regulates dendritic cell cross-presentation and T cell responses by inhibiting receptor-mediated antigen uptake. *J. Immunol.* 188 5489–5499. 10.4049/jimmunol.1103490 22544935 PMC3358354

[B21] Guo J. Cui L. Lu Q. Zhang Y. Liu Q. Wang X. (2018). Cyadox regulates the transcription of different genes by activation of the PI3K signaling pathway in porcine primary hepatocytes. *J. Cell. Biochem.* 10.1002/jcb.28037 [Epub ahead of print]. 30417433

[B22] Huang Y. Zhang J. Hou L. Wang G. Liu H. Zhang R. (2017). LncRNA AK023391 promotes tumorigenesis and invasion of gastric cancer through activation of the PI3K/Akt signaling pathway. *J. Exp. Clin. Cancer Res.* 36:194. 10.1186/s13046-017-0666-2 29282102 PMC5745957

[B23] Kasprowicz D. J. Smallwood P. S. Tyznik A. J. Ziegler S. F. (2003). Scurfin (FoxP3) controls T-dependent immune responses in vivo through regulation of CD4+ T cell effector function. *J. Immunol.* 171 1216–1223. 12874208 10.4049/jimmunol.171.3.1216

[B24] Kong L. Zhang Y. Ye Z. Q. Liu X. Q. Zhao S. Q. Wei L. (2007). CPC: assess the protein-coding potential of transcripts using sequence features and support vector machine. *Nucleic Acids Res.* 35 W345–W349. 10.1093/nar/gkm391 17631615 PMC1933232

[B25] Kopp F. Mendell J. T. (2018). Functional classification and experimental dissection of long noncoding RNAs. *Cell* 172 393–407. 10.1016/j.cell.2018.01.011 29373828 PMC5978744

[B26] Kramer W. Girbig F. Corsiero D. Pfenninger A. Frick W. Jahne G. (2005). Aminopeptidase N (CD13) is a molecular target of the cholesterol absorption inhibitor ezetimibe in the enterocyte brush border membrane. *J. Biol. Chem.* 280 1306–1320. 10.1074/jbc.M406309200 15494415

[B27] Kwok Z. H. Tay Y. (2017). Long noncoding RNAs: lincs between human health and disease. *Biochem. Soc. Trans.* 45 805–812. 10.1042/bst20160376 28620042

[B28] Lemler D. J. Brochu H. N. Yang F. Harrell E. A. Peng X. (2017). Elucidating the role of host long non-coding rna during viral infection: challenges and paths forward. *Vaccines* 5:37. 10.3390/vaccines5040037 29053596 PMC5748604

[B29] Li X. Wu Z. Fu X. Han W. (2014). lncRNAs: insights into their function and mechanics in underlying disorders. *Mutat. Res. Rev. Mutat. Res.* 762 1–21. 10.1016/j.mrrev.2014.04.002 25485593

[B30] Li Z. Li X. Jiang C. Qian W. Tse G. Chan M. T. V. (2018). Long non-coding RNAs in rheumatoid arthritis. *Cell Prolif.* 51:e12404. 10.1111/cpr.12404 29110355 PMC6620844

[B31] Liang H. Zen K. Zhang J. Zhang C. Y. Chen X. (2013). New roles for microRNAs in cross-species communication. *RNA Biol.* 10 367–370. 10.4161/rna.23663 23364352 PMC3672279

[B32] Liu Q. Lei Z. Zhou K. Yu H. Liu S. Sun Q. (2018). N-O reduction and ROS-Mediated AKT/FOXO1 and AKT/P53 pathways are involved in growth promotion and cytotoxicity of cyadox. *Chem. Res. Toxicol.* 31 1219–1229. 10.1021/acs.chemrestox.8b00194 30265530

[B33] Matsushima Y. Ishikawa O. Kurosawa M. Miyachi Y. (2000). Stem cell factor and IL-6 do not promote complete maturation of human cultured mast cells from umbilical cord blood cells: an ultrastructural study. *J. Dermatol. Sci.* 24 4–13. 10960774 10.1016/s0923-1811(00)00076-1

[B34] Metz M. Maurer M. (2007). Mast cells–key effector cells in immune responses. *Trends Immunol.* 28 234–241. 10.1016/j.it.2007.03.003 17400512

[B35] Moriggl R. Topham D. J. Teglund S. Sexl V. Mckay C. Wang D. (1999). Stat5 is required for IL-2-induced cell cycle progression of peripheral T cells. *Immunity* 10 249–259. 10.1016/S1074-7613(00)80025-4 10072077

[B36] Mowel W. K. Kotzin J. J. Mccright S. J. Neal V. D. Henao-Mejia J. (2018). Control of immune cell homeostasis and function by lncRNAs. *Trends Immunol.* 39 55–69. 10.1016/j.it.2017.08.009 28919048 PMC5748345

[B37] Nakajima H. Liu X.-W. Wynshaw-Boris A. Rosenthal L. A. Imada K. Finbloom D. S. (1997). An indirect effect of stat5a in IL-2–induced proliferation: a critical role for Stat5a in IL-2–mediated IL-2 receptor α chain Induction. *Immunity* 7 691–701. 10.1016/S1074-7613(00)80389-1 9390692

[B38] Nefla M. Sudre L. Denat G. Priam S. Andre-Leroux G. Berenbaum F. (2015). The pro-inflammatory cytokine 14-3-3epsilon is a ligand of CD13 in cartilage. *J. Cell. Sci.* 128 3250–3262. 10.1242/jcs.169573 26208633 PMC4582189

[B39] Noh J. H. Kim K. M. Mcclusky W. G. Abdelmohsen K. Gorospe M. (2018). Cytoplasmic functions of long noncoding RNAs. *Wiley Interdiscip. Rev. RNA* 9:e1471. 10.1002/wrna.1471 29516680 PMC5963534

[B40] Ono M. Yaguchi H. Ohkura N. Kitabayashi I. Nagamura Y. Nomura T. (2007). Foxp3 controls regulatory T-cell function by interacting with AML1/Runx1. *Nature* 446 685–689. 10.1038/nature05673 17377532

[B41] Reinhold D. Biton A. Goihl A. Pieper S. Lendeckel U. Faust J. (2007). Dual inhibition of dipeptidyl peptidase IV and aminopeptidase N suppresses inflammatory immune responses. *Ann. N.Y. Acad. Sci.* 1110 402–409. 10.1196/annals.1423.042 17911455

[B42] Roberts A. P. Lewis A. P. Jopling C. L. (2011). The role of microRNAs in viral infection. *Prog. Mol. Biol. Transl. Sci.* 102 101–139. 10.1016/b978-0-12-415795-8.00002-7 21846570

[B43] Rosenberger C. M. Podyminogin R. L. Navarro G. Zhao G. W. Askovich P. S. Weiss M. J. (2012). miR-451 regulates dendritic cell cytokine responses to influenza infection. *J. Immunol.* 189 5965–5975. 10.4049/jimmunol.1201437 23169590 PMC3528339

[B44] Scott M. E. Ma Y. Kuzmich L. Moscicki A. B. (2009). Diminished IFN-gamma and IL-10 and elevated Foxp3 mRNA expression in the cervix are associated with CIN 2 or 3. *Int. J. Cancer* 124 1379–1383. 10.1002/ijc.24117 19089920 PMC2696072

[B45] Selliah N. Zhang M. Desimone D. Kim H. Brunner M. Ittenbach R. F. (2006). The gammac-cytokine regulated transcription factor, STAT5 increases HIV-1 production in primary CD4 T cells. *Virology* 344 283–291. 10.1016/j.virol.2005.09.063 16289657

[B46] Shi L. Zhang N. Liu H. Zhao L. Liu J. Wan J. (2018). Lysyl oxidase inhibition via beta-aminoproprionitrile hampers human umbilical vein endothelial cell angiogenesis and migration in vitro. *Mol. Med. Rep.* 17 5029–5036. 10.3892/mmr.2018.8508 29393489 PMC5865964

[B47] Soderberg C. Giugni T. D. Zaia J. A. Larsson S. Wahlberg J. M. Moller E. (1993). CD13 (human aminopeptidase N) mediates human cytomegalovirus infection. *J. Virol.* 67 6576–6585. 8105105 10.1128/jvi.67.11.6576-6585.1993 PMC238095

[B48] Thomas R. Davis L. S. Lipsky P. E. (1994). Rheumatoid synovium is enriched in mature antigen-presenting dendritic cells. *J. Immunol.* 152 2613–2623. 7510751

[B49] Triulzi T. Tagliabue E. Balsari A. Casalini P. (2013). FOXP3 expression in tumor cells and implications for cancer progression. *J. Cell Physiol.* 228 30–35. 10.1002/jcp.24125 22674548

[B50] Wang H. Peng W. Ouyang X. Li W. Dai Y. (2012). Circulating microRNAs as candidate biomarkers in patients with systemic lupus erythematosus. *Transl. Res.* 160 198–206. 10.1016/j.trsl.2012.04.002 22683424

[B51] Wang X. Liu Q. Ihsan A. Huang L. Dai M. Hao H. (2012). JAK/STAT pathway plays a critical role in the proinflammatory gene expression and apoptosis of RAW264.7 cells induced by trichothecenes as DON and T-2 toxin. *Toxicol. Sci.* 127 412–424. 10.1093/toxsci/kfs106 22454431

[B52] Wang P. Xue Y. Han Y. Lin L. Wu C. Xu S. (2014). The STAT3-binding long noncoding RNA lnc-DC controls human dendritic cell differentiation. *Science* 344 310–313. 10.1126/science.1251456 24744378

[B53] Wang Z. Xi J. Hao X. Deng W. Liu J. Wei C. (2017). Red blood cells release microparticles containing human argonaute 2 and miRNAs to target genes of Plasmodium falciparum. *Emerg. Microb. Infect* 6:e75. 10.1038/emi.2017.63 28831191 PMC5583671

[B54] Wu Z. Liu X. Liu L. Deng H. Zhang J. Xu Q. (2014). Regulation of lncRNA expression. *Cell Mol. Biol. Lett.* 19 561–575. 10.2478/s11658-014-0212-6 25311814 PMC6275606

[B55] Yamada H. Itoh M. Hiratsuka I. Hashimoto S. (2014). Circulating microRNAs in autoimmune thyroid diseases. *Clin. Endocrinol.* 81 276–281. 10.1111/cen.12432 24533739

[B56] Yang X. P. Ghoreschi K. Steward-Tharp S. M. Rodriguez-Canales J. Zhu J. Grainger J. R. (2011). Opposing regulation of the locus encoding IL-17 through direct, reciprocal actions of STAT3 and STAT5. *Nat. Immunol.* 12 247–254. 10.1038/ni.1995 21278738 PMC3182404

[B57] Yu R. Zhang Y. Lu Q. Cui L. Wang Y. Wang X. (2018). Differentially expressed genes in response to cyadox in swine liver analyzed by DDRT-PCR. *Res. Vet. Sci.* 118 72–78. 10.1016/j.rvsc.2018.01.014 29421487

[B58] Zhang J. Zhou Y. Wu Y. Ma L. Fan Y. Kang X. (2012). Isolation and characterization of a novel noncoding RNA from nickel-induced lung cancer. *Biol. Trace Elem. Res.* 150 258–263. 10.1007/s12011-012-9460-3 22665269

[B59] Zotz J. S. Wolbing F. Lassnig C. Kauffmann M. Schulte U. Kolb A. (2016). CD13/aminopeptidase N is a negative regulator of mast cell activation. *FASEB J.* 30 2225–2235. 10.1096/fj.201600278 26936360

