# Supplementary material for: Molecular Characterization and Biological Function of a Novel LncRNA CRNG in Swine
Source: Front Pharmacol. 2019 May 21;10:539. doi: 10.3389/fphar.2019.00539 (PMC6537671; doi:10.3389/fphar.2019.00539)
Supplement: DATA SHEET S1 — The result of comparing CRNG with NCBI database. [file Data_Sheet_1.PDF]

```
Tue Oct 23, 2018 21:44 CST
953.ape from 1 to 953
Alignment to
76513882-76516982.ape from 1 to 3101

Matches(|):951
Mismatches(#):1
Gaps( ):2150
Unattempted(.):0

               *         *         *         *         *         *         *         *         *         *
    1 ACAGCCTCCTGTCTCGGTGGTTGTCGTGGCCCTGACGCCCTCACTCCCACAAGAAGAAGACACAGCGAAGGAGAAGGGACGGCCCCTCAGGGCGGCTTCT 100
      ||||||||||||||||||||||||||||||||||||||||||||||||||||||||||||||||||||||||||||||||||||||||||||||||||||
    1 ACAGCCTCCTGTCTCGGTGGTTGTCGTGGCCCTGACGCCCTCACTCCCACAAGAAGAAGACACAGCGAAGGAGAAGGGACGGCCCCTCAGGGCGGCTTCT 100
               *         *         *         *         *         *         *         *         *         *

               *         *         *         *         *         *         *         *         *         *
  101 GGGCGCGGATGTGCCTGAAATAGTCAACTGAACGGAGGCAGCACCGAGGGGGGGCGACGAGGAAGGAGGCCCAGGGTGCGTGATAATCACGGATGCGGAG 200
      ||||||||||||||||||||||||||||||||||||||||||||||||||||||||||||||||||||||||||||||||||||||||||||||||||||
  101 GGGCGCGGATGTGCCTGAAATAGTCAACTGAACGGAGGCAGCACCGAGGGGGGGCGACGAGGAAGGAGGCCCAGGGTGCGTGATAATCACGGATGCGGAG 200
               *         *         *         *         *         *         *         *         *         *

               *         *         *         *         *         *         *         *         *         *
  201 ACTCACTCACTCGGGGGCGGACACCTGGCCCCCCCAGTCTGACACCAAGGAGTAAACAAAACCCCCCCCAACCTAGAGAGGCCCCCAAACGCCACCCAGC 300
      |||||||||||||||||||||||||||||||||||||||||||||||||||||||||||||||||||| |||||||||||||||||||||||||||||||
  201 ACTCACTCACTCGGGGGCGGACACCTGGCCCCCCCAGTCTGACACCAAGGAGTAAACAAAACCCCCCC-AACCTAGAGAGGCCCCCAAACGCCACCCAGC 299
               *         *         *         *         *         *          *         *         *

               *         *         *         *         *         *         *         *         *         *
  301 TCTCTTGCGACTGCCACTTATTTTTGCACCGTGTGCCCTTCTCCTGCGTGTCCTGGGCTCTTTCGCAAACCTGTTGATCGAATGTATGGAACATTTCTGT 400
      ||||||||||||||||#|||||||||||||||||||||||||||||||||||||||||||||||||||||||||||||||||||||||||||||||||||
  300 TCTCTTGCGACTGCCATTTATTTTTGCACCGTGTGCCCTTCTCCTGCGTGTCCTGGGCTCTTTCGCAAACCTGTTGATCGAATGTATGGAACATTTCTGT 399
      *         *         *         *         *         *         *         *         *         *

               *         *         *         *         *         *         *         *         *         *
  401 TCACGACGGCTGAGGATTAATGATTCACCAAGGACACAGGCCCTTTGTACCTATAATTGTCCTGCATTTGTTTGGACTCTCTTTGACCCTTTGTACCCAA 500
      ||||||||||||||||||||||||||||||||||||||||||||||||||||||||||||||||||||||||||||||||||||||||||||||||||||
  400 TCACGACGGCTGAGGATTAATGATTCACCAAGGACACAGGCCCTTTGTACCTATAATTGTCCTGCATTTGTTTGGACTCTCTTTGACCCTTTGTACCCAA 499
      *         *         *         *         *         *         *         *         *         *

               *         *         *         *         *         *         *         *         *         *
  501 GTGGTCTGTGGTGGAGCCCTTTGAAAACAATCTCCTGGATAAAGTGTTTATGGGATTTACCGCAAGCCCGTGGCTCTTTTGCTGTGACGCCTGCTGCGTA 600
      ||||||||||||||||||||||||||||||||||||||||||||||||||||||||||||||||||||||||||||||||||||||||||||||||||||
  500 GTGGTCTGTGGTGGAGCCCTTTGAAAACAATCTCCTGGATAAAGTGTTTATGGGATTTACCGCAAGCCCGTGGCTCTTTTGCTGTGACGCCTGCTGCGTA 599
      *         *         *         *         *         *         *         *         *         *

               *         *         *         *         *         *         *         *         *
  601 AGAGAGACCAAAACCACCATCTCAACGAGGTCAAACGTGCCGTCACCCCAAGAGCGAGACATTCCTCCTGGGTCCTCCGTAAGCGCCCCG---------- 690
      ||||||||||||||||||||||||||||||||||||||||||||||||||||||||||||||||||||||||||||||||||||||||||
  600 AGAGAGACCAAAACCACCATCTCAACGAGGTCAAACGTGCCGTCACCCCAAGAGCGAGACATTCCTCCTGGGTCCTCCGTAAGCGCCCCGAGGTGAGATA 699
      *         *         *         *         *         *         *         *         *         *

  691 ---------------------------------------------------------------------------------------------------- 691

  700 TTAAATATTATTTTGAGCAGTGGATGGCCCATCTGTGCCCAAGGGCTGTTACACAAAGTCTGCCTCTCTGCAGAGAACAAGGGTCGGCTGGCCCCAGCTC 799
      *         *         *         *         *         *         *         *         *         *

  691 ---------------------------------------------------------------------------------------------------- 691

  800 TGTCCTCATTTTTGCTCTGAGCAGGACGGTCAAAGTTCTCGGCAGGTACATCTCGAGGGGCTCTGTCTATACCTCTGATCACGTAGTGACCGAGGAGCCC 899
      *         *         *         *         *         *         *         *         *         *

  691 ---------------------------------------------------------------------------------------------------- 691

  900 AGCTTCCCACCCAACAGCTCAGGTCAAGAGGCAGGACAGAATGTGTCCCCGTGTCGGTTTCCTCTGTCACCCCTCGGACACCTCTTAGCGGAGTGACCGC 999
      *         *         *         *         *         *         *         *         *         *

  691 ---------------------------------------------------------------------------------------------------- 691

 1000 CTCGGCCTTCCTCTCCTCGTCTCCGTTCACACCATCCGCAAACATGCACTGCGGGGCTGCCCTCGGGGAAGCGCCTTGCAGAAATCAAGCAGGGTGACCT 1099
      *         *         *         *         *         *         *         *         *         *
```

```
 691 ---------------------------------------------------------------------------------------------------- 691

1100 GTGAATTCCAGGAATTCCTAATCTCGCGCTGGGCATGGACATGGCACATCCACTTTCAAAACAAAGTTGTCAAGGTGCATGCCACGTGTCTGATACCAAG 1199
     *         *         *         *         *         *         *         *         *         *

 691 ---------------------------------------------------------------------------------------------------- 691

1200 AACTGTGGCTCGGGGTTCGGGGGAGGGTCCTAGCCTTGTACTCTCTTATCCAGAGACCACTTCGCAAAGCTGGTAGATGTGGGCTGGTGGAATGAATATG 1299
     *         *         *         *         *         *         *         *         *         *

 691 ---------------------------------------------------------------------------------------------------- 691

1300 GTGACGGGCGGACGATGGCAGGATGAGGCGGTGGAGCCAGTGAATCGGGGAGTGGGGAATTCACTCAGAGAGTCCGGCCAGACAGGGGAGTTCAAGGTCT 1399
     *         *         *         *         *         *         *         *         *         *

 691 ---------------------------------------------------------------------------------------------------- 691

1400 GCATTAATTGGAAGCATCACCGAGGCTAGCGGTGATGAGGGTGTCTTATTTACTATGAGTCAGGATGAGCTGTCACAGGAAGAGAAGAAGATGCCGCACT 1499
     *         *         *         *         *         *         *         *         *         *

 691 ---------------------------------------------------------------------------------------------------- 691

1500 ACCAGGCAAAAAACTGCAATCACTGCTTCAACTCTAGCAGTAAGACTTAGCAGGCTGCTCAGATTTCGAGCTGGAAAACGTGCTGCGATGTTGACAAATA 1599
     *         *         *         *         *         *         *         *         *         *

 691 ---------------------------------------------------------------------------------------------------- 691

1600 TTATCATGTTTTCATACCCTTTTCTTTAAGAACAGCCCCGGGAGAAGTCTGACATAGCGGTCTCTTCCTGGTCTCAGAGTGTGCAAATCCATGTACAAAG 1699
     *         *         *         *         *         *         *         *         *         *

 691 ---------------------------------------------------------------------------------------------------- 691

1700 ACCCGGAGGGAATTCTCAAAACGTTTAAATATCAATTGATTCGACCATCATTTCTGCCCTCCTGGGCTGTGAACTCTGACCTCATAAAGACAGGCTGATG 1799
     *         *         *         *         *         *         *         *         *         *

 691 ---------------------------------------------------------------------------------------------------- 691

1800 AAGCCAACAGCTGGTCTGAACCAAGTCACCACTTGTCAGTTACTGTAAACAAAGTCCCATGATGAGCTTCGCGCTCTTTAAATCGATCAATTACCAGATT 1899
     *         *         *         *         *         *         *         *         *         *

 691 ---------------------------------------------------------------------------------------------------- 691

1900 CTAACAGCCATAAACTTTGCCAATTCAATACAACGGGACAAGACGGCTGCTTTCTGTTTAAAAATATTTTCCAAATAATCAGAAACCGCATTCCCCATGG 1999
     *         *         *         *         *         *         *         *         *         *

 691 ---------------------------------------------------------------------------------------------------- 691

2000 TCCCCCCCCGCCCCCCCGCCAAAAGCAATGCATGTTTCCCTACTTGGGAAATTGTTTATTGTCAAGTACCTATATTCTAAGGGACTATTGGTTCTCCTGG 2099
     *         *         *         *         *         *         *         *         *         *

 691 ---------------------------------------------------------------------------------------------------- 691

2100 CCTCGTGTAGGAAAACCAACAGGTCAAGATTGGTGAAAAGTCTCATGTTGACTCAACATAACTTTATCTCCTATAAAAGCATTTTCTAACTGCAAGCTAT 2199
     *         *         *         *         *         *         *         *         *         *

 691 ---------------------------------------------------------------------------------------------------- 691

2200 AACAGACCTAGGGTCACGCCACAATAATGACTTTGCAAAACACTCTTAGAAAACACTGTTACTGGTTTTGGTTTGAGGGGCTGCAGCCCGTGGCCCGTGC 2299
     *         *         *         *         *         *         *         *         *         *

 691 ---------------------------------------------------------------------------------------------------- 691

2300 CACCAGGTTTAAAGTTCTTTGCATGCCTGAGGATGGAGCTGGGAGACGAGAGAACATGCGGGACCTACTCTGCACAGAAACCAGGGCACGGGTGTTTGTG 2399
     *         *         *         *         *         *         *         *         *         *

 691 ---------------------------------------------------------------------------------------------------- 691

2400 AATCATTTCAGTCCTTAAAAATGTTTCCAGGAGCCCCTTTGTGGCTCAGCGGGTTAAGAATCCGGCATTGTCACTGCTGCGGCTGGAGTTGCTGCTGGGG 2499
     *         *         *         *         *         *         *         *         *         *
```

```
 691 ---------------------------------------------------------------------------------------------------- 691

2500 CGTGGGTTTGATCCCTGGCCCCAGAAATTCCATATGCTGCAGGCACGGTCAAAAAAAAAAAAAAAAAAAATCCAAATTCCAATATCATTACATTGAAGGCA 2599
     *         *         *         *         *         *         *         *         *         *

 691 ---------------------------------------------------------------------------------------------------- 691

2600 AAGGAAGAAGCTAATTTTAGAAGCTCCCTGGGGTCCTGCTCTGACACCCACATGTCCAAATCCCACGCTTCTGCATGCACGTGTTTACTACACACCCTCA 2699
     *         *         *         *         *         *         *         *         *         *

 691 ---------------------------------------------------------------------------------------------------- 691

2700 TGAACCACTTACCAAGGGTTCCACCGTGAGCTCTTCCTTCAAATTGTATTATCGCAGAGATAAAAGGAGATTTGTGTTATGGCGGTTGAAGGCGCCCATT 2799
     *         *         *         *         *         *         *         *         *         *

                                                    *         *         *         *         *         *
 691 --------------------------------------AGGCATCCCAGGCGCTGGAACATAAAGAAGATGAGAAACGCTCTCTCAGAGAACTTGCTTCC 752
                                           ||||||||||||||||||||||||||||||||||||||||||||||||||||||||||||||
2800 CCGAAAGTGATAACCCATTTAATCCTTCCTTCAACTACAGGCATCCCAGGCGCTGGAACATAAAGAAGATGAGAAACGCTCTCTCAGAGAACTTGCTTCC 2899
     *         *         *         *         *         *         *         *         *         *

            *         *         *         *         *         *         *         *         *         *
 753 TGGTCTCGGCTACACCCCAGACTCTCAGAGTCTAGTCTGAGTCTATACGCATCTGTGCTTAGGATCACTCGGCATGGGTTCGAGTAAGCCCATCTGTGAG 852
     ||||||||||||||||||||||||||||||||||||||||||||||||||||||||||||||||||||||||||||||||||||||||||||||||||||
2900 TGGTCTCGGCTACACCCCAGACTCTCAGAGTCTAGTCTGAGTCTATACGCATCTGTGCTTAGGATCACTCGGCATGGGTTCGAGTAAGCCCATCTGTGAG 2999
     *         *         *         *         *         *         *         *         *         *

            *         *         *         *         *         *         *         *         *         *
 853 AGCCTTGTAAAAACATATCTATTGTCATTCCGATTCATCTTATGGATGAGCCCCTTCCGATGTTTGAAAACATGAAATTCCAACACATGTGAAAAAAATC 952
     ||||||||||||||||||||||||||||||||||||||||||||||||||||||||||||||||||||||||||||||||||||||||||||||||||||
3000 AGCCTTGTAAAAACATATCTATTGTCATTCCGATTCATCTTATGGATGAGCCCCTTCCGATGTTTGAAAACATGAAATTCCAACACATGTGAAAAAAATC 3099
     *         *         *         *         *         *         *         *         *         *


 953 A~ 953
     |
3100 AC 3101
     *
```
