# Supplementary material for: Molecular Characterization and Biological Function of a Novel LncRNA CRNG in Swine
Source: Front Pharmacol. 2019 May 21;10:539. doi: 10.3389/fphar.2019.00539 (PMC6537671; doi:10.3389/fphar.2019.00539)
Supplement: DATA SHEET S3 — The silencing or overexpression efficiency after transfecting the cells with siRNA or CRNG- expression vector, the silencing efficiency of CRNG was showed in Figure 2 and CRNG induced by cyadox in PK-15 cells was shown in Figure 3. The siRNA sequences of CRNG were showed in Table 1. [file Data_Sheet_3.pdf]

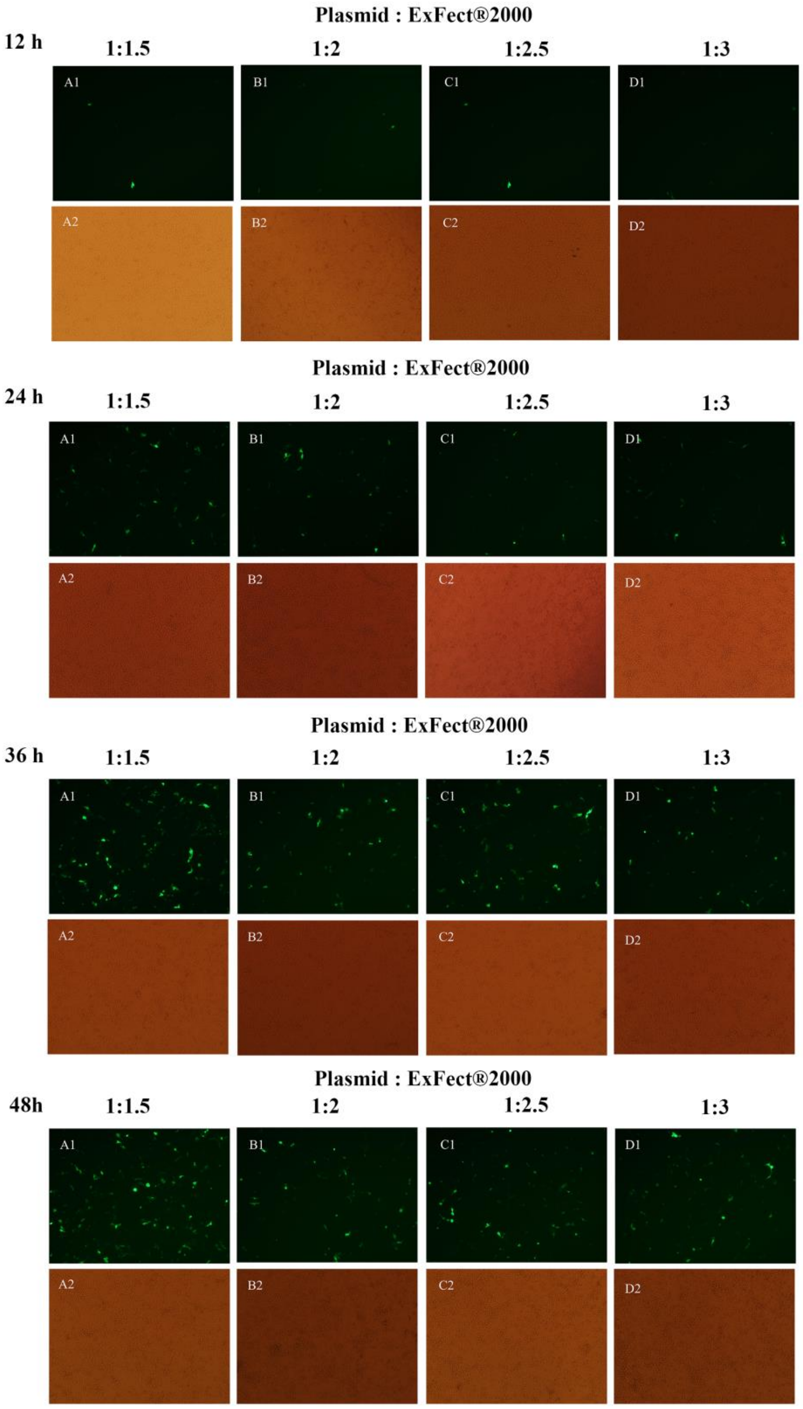

Figure 1 The overexpression efficiency after transfecting the cells with pcDNA3.0-EGFP-CRNG vector

Note: According to the effect of transfection, Plasmid: ExFect®2000=1: 1.5 (μg/μL) was selected and transfected for 48 h.

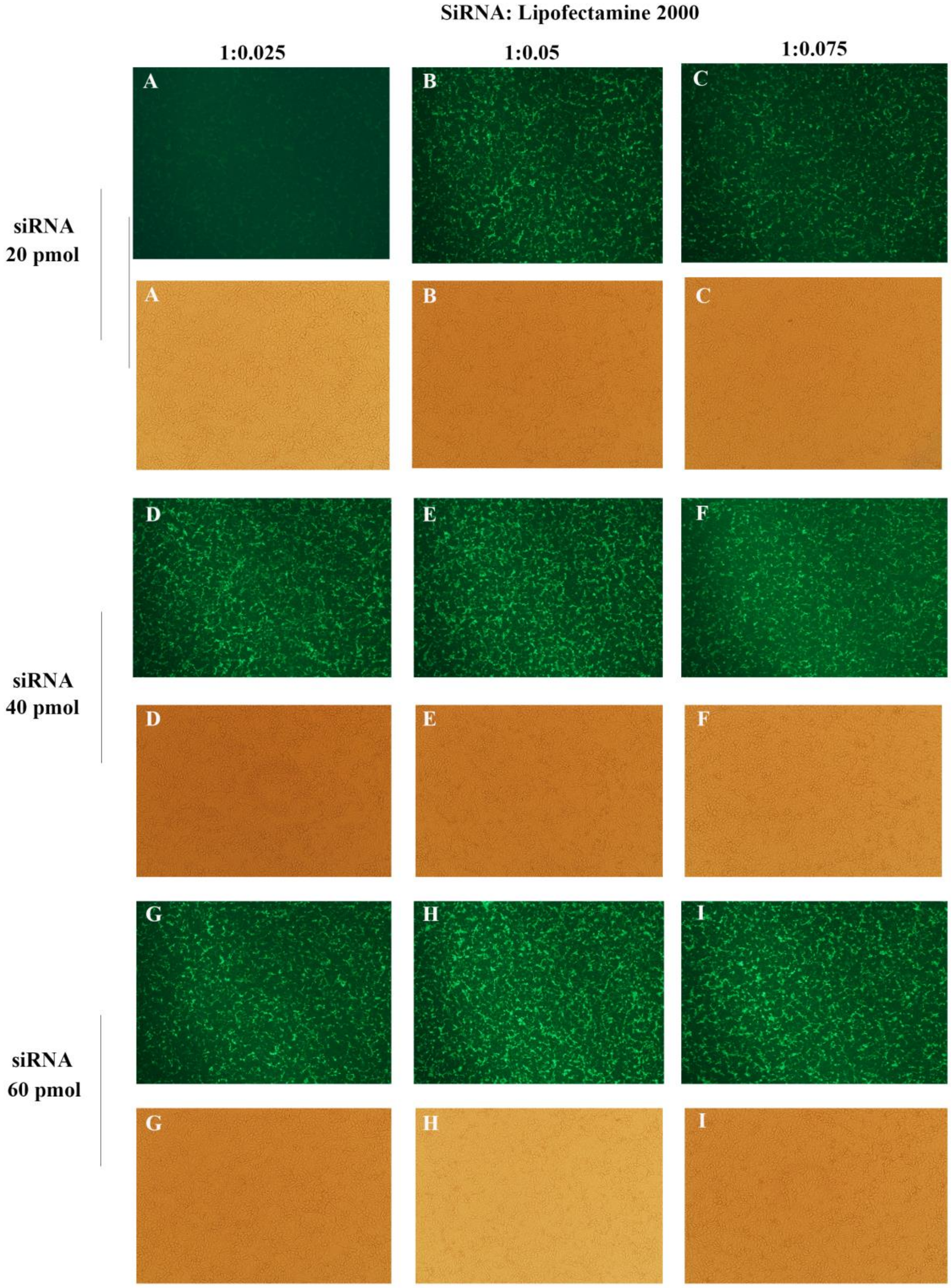
SiRNA: Lipofectamine 2000
1:0.025
1:0.05
1:0.075
siRNA
20 pmol
siRNA
40 pmol
siRNA
60 pmol
A
B
C
D
E
F
G
H
I

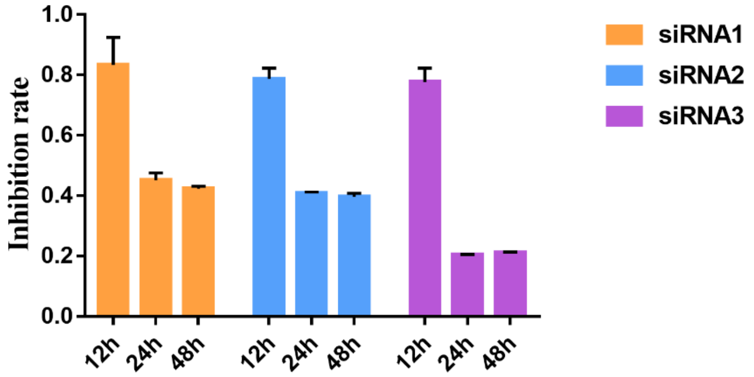

Figure 2 The silencing efficiency after transfecting the cells with siRNA

Note: Negative control FAM (UUCUCCGAACGUGUCACGUTT; ACGUGACACGUUCGGAGAATT). According to the effect of transfection, siRNA FAM 40 pmol and siRNA: Lipofectamine 2000 was selected.

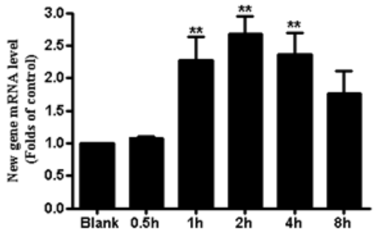

Figure 3 The time-effect relationships of CRNG induced by cyadox in PK-15 cells

Table 1 The siRNA sequence of CRNG

Name	Sequence（5’- 3’）
Negative control	UUCUCCGAACGGUCACGUTT
	ACGUGACACGUUCGGAGAATT
Negative control FAM	UUCUCCGAACGUGUCACGUTT
	ACGUGACACGUUCGGAGAATT
siRNA1	GCAAACCUGUUGAUCGAAUTT
	AUUCGAUCAACAGGUUUGCTT
siRNA2	GGACUCUCUUUGACCCUUUTT
	AAAGGGUCAAAGAGAGUCCTT
siRNA3	GCAUCUGUGCUUAGGAUCATT
	UGAUCCUAAGCACAGAUGCTT
